# Zero-shot transfer learned generic AI models for prediction of optimally ripe climacteric fruits

**DOI:** 10.1038/s41598-023-34527-8

**Published:** 2023-05-05

**Authors:** Jayita Dutta, Manasi Patwardhan, Parijat Deshpande, Shirish Karande, Beena Rai

**Affiliations:** Physical Sciences Research Area, Tata Research Development and Design Centre, TCS Research, Tata Consultancy Services, 54, B Hadapsar Industrial Estate, Pune, 411013 India

**Keywords:** Biochemistry, Statistics, Environmental impact

## Abstract

Ideally, ripe fruits offer appropriate nutritional content and best quality in terms of taste and flavour. Prediction of ripe climacteric fruits acts as the main marketing indicator for quality from the consumer perspective and thus renders it a genuine industrial concern for all the stakeholders of the fruit supply chain. However, the building of fruit-specific individual model for the prediction of ripeness level remains an existing challenge due to the scarcity of sufficient labeled experimental data for each fruit. This paper describes the development of generic AI models based on the similarity in physico-chemical degradation phenomena of climacteric fruits for prediction of ‘unripe’ and ‘ripe’ levels using ‘zero-shot’ transfer learning techniques. Experiments were performed on a variety of climacteric and non-climacteric fruits, and it was observed that transfer learning works better for fruits within a cluster (climacteric fruits) as compared to across clusters (climacteric to non-climacteric fruits). The main contributions of this work are two-fold (i) Using domain knowledge of food chemistry to label the data in terms of age of the fruit, (ii) We hypothesize and prove that the zero-shot transfer learning works better within a set of fruits, sharing similar degradation chemistry depicted by their visual properties like black spot formations, wrinkles, discoloration, etc. The best models trained on banana, papaya and mango dataset resulted in s zero-shot transfer learned accuracies in the range of 70 to 82 for unknown climacteric fruits. To the best of our knowledge, this is the first study to demonstrate the same.

## Introduction

Ripening of fruits is a complex physico-chemical phenomenon resulting in various physiological, biochemical and developmental changes in a coordinated and genetically regulated manner^1–3^. These changes account for the perfect colour, texture, flavour, and aroma of ripe fruits^4^. Fruits can further be classified into climacteric and non-climacteric based on their respiratory pattern during ripening^2^. Climacteric fruits such as banana, papaya, mango, apple, peach, pear and avocado ripen post-harvest and can be characterized by an increase in CO_2_ respiration rate and ethylene emission rate. As climacteric fruits ripen post-harvest, a parallel increase in respiration rate and ethylene emission rate is observed, followed by an ethylene-assisted peak called the ‘climacteric peak’. Once the climacteric peak is reached, there is a decrease in ethylene emission rate and respiration rate analogous to the non-climacteric fruits. Unlike climacteric fruits, non-climacteric fruits such as lemon, orange, litchi, cherry and strawberry are ripe when harvested and are characterized by the absence of ethylene-assisted climacteric peak^2–6^.

Consumers prefer optimally ripe fruits with optimal nutritional content and the best taste and flavour^7^. Often, stakeholders involved in the fruit supply chain faces sales obstacles from the customer if the fruits are not optimally ripened for consumption. Thereby, all the stakeholders face a hard time in clearing up the stocks of fruits within the required time, therefore, resulting in huge wastage of fruits across the supply chain and ends up in economic losses and also causes a hindrance to the achievement of sustainable development goal (SDGs). Thus, it is a genuine industrial necessity for the stakeholders of the fruit supply chain to be able to predict the ideal ripeness level of fruits^8–10^.

Fruits are considered fully ripe when they attain a desirable colour, texture, flavour and aroma. These desirable properties account for changes in the chemical composition in terms of, starch to sugar conversion, followed by an increase in sugar content and reduction in organic acids, and changes^11^. All climacteric fruits exhibit the ethylene-assisted climacteric peak in their post-harvest maturation phase when they are optimally ripe with best taste and flavour. Unlike climacteric fruits, non-climacteric fruits are expected to be fully ripe when harvested with ideal sugar content, taste and flavour^5^.

Various bespoke models have also been developed in this regard over the years for monitoring of ripeness level of fruits^12–17^. However, the building of fruit specific bespoke model for the prediction of ideal ripeness level of fruits is extremely challenging as it would require a large amount of annotated data for each fruit.

Moreover, most of these methods used for monitoring of ripeness level of fruits involve extensive invasive wet-lab experiments or rely on judgement of food domain experts for annotation of experimental data, thus making the process much more tedious, time-consuming, and costly^18–21^. Furthermore, logical analysis suggests accurate prediction of the age of fruits is difficult even for the experts just by looking at the images. Also, image annotations by performing invasive lab experiments and applying food chemistry knowledge are only possible for a small set of images. Most importantly, when real-life supply chain scenarios are considered, performing invasive testing in real-time for the prediction of ripeness level of fruits seems unlikely.

This paper focuses on the development of convolution neural network (CNN)^22^ based AI models for the prediction of ripeness levels of climacteric fruits using ‘zero-shot’ transfer learning techniques^23^. Zero-shot transfer learning techniques have been used previously for various text-based applications such as event extraction, sentiment analysis, sign and alphabet recognition^23–27^ etc. However, to the best of our knowledge, the same was not used in food domain for quality prediction task. In this paper, the similarities in physico-chemical degradation patterns of climacteric fruits were exhausted for transfer of knowledge from fruits with adequate labeled experimental data such as banana, papaya, and mango to fruits with limited data such as apple, peach, pear, and avocado. CNN-based models were also trained on non-climacteric fruit lemon and were used to predict the ripeness level of climacteric fruits to validate if zero-shot transfer of knowledge was possible from climacteric to non-climacteric fruits. In synch with the known degradation chemistry of fruits^5,11^, the models demonstrated that transfer learning proved beneficial within climacteric fruits and was weak from climacteric to non-climacteric fruits and vice-versa. Once deployed, these models trained with small annotated data in a zero-shot setting acts as soft sensors and provides a cost-effective, non-invasive, real-time solution to the stakeholders of the fruit supply chain for the prediction of ripeness level of climacteric and non-climacteric fruits. The developed models can take the image of a fruit as an input which can be easily captured by a smartphone camera and predict the ripeness level of the fruit. This model can easily reside either on an android based smartphone (a distilled version that take lesser memory) or on the cloud, thus making it convenient for the stakeholders to take dynamic decisions in real-time based on the ripeness level of the fruit.

## Materials and methods

### Materials

Ripe and unripe samples of climacteric fruits namely ‘Kesar’ cultivar of mangoes (scientific name: *Mangifera indica* 'Gir Kesar'), ‘Cavendish’ cultivar of bananas (scientific name: *Musa acuminata* ‘Cavendish Subgroup’), and ‘Pusa Majesty’ cultivar of papayas (scientific name: *Carica papaya* 'Pusa Majesty'), and non-climacteric fruits namely ‘Nepali Oblong’ cultivar of lemons (scientific name: *Citrus limon* 'Nepali Oblong') were used for experimental analysis. Banana, papaya and lemon cultivars were grown and harvested at a farm near Udhali Bk, Tal-Raver, Jalgaon district, Maharashtra. The mango cultivars were grown and harvested from a farm at a farm in Latur district of Marathwada region of Maharashtra. Banana, papaya, mango and lemon were harvested after 130, 125, 120, 160 days of blossoming respectively. The fruits were grown organically without any chemicals.

### Experimental methods

**Figure 1:**
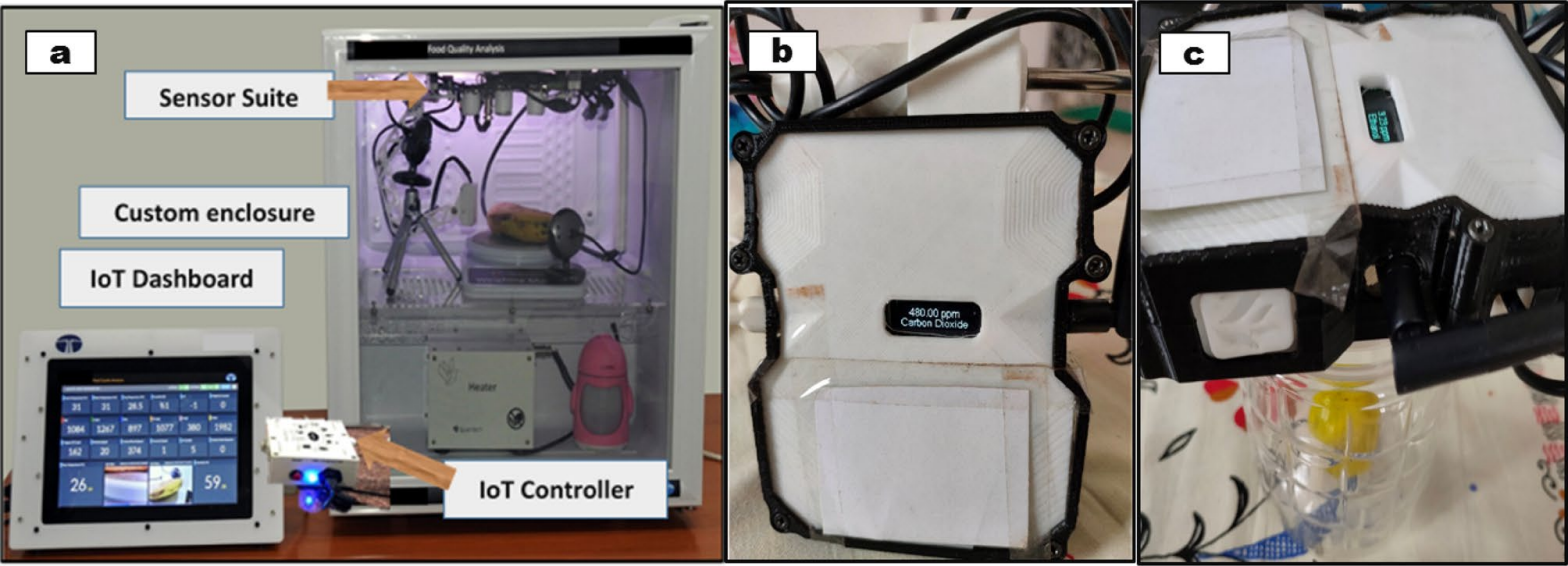
(**a**) IoT-enabled experimental setup developed (**b**) handheld device with ethylene sensor and camera (**c**) handheld device used to measure variation in ethylene rate and images of fruits kept inside a transparent plastic beaker.

A temperature and humidity controlled custom enclosure of 40 L volume with provisions for controlled ventilation was developed for labelling fruit images as ‘unripe’ and ‘ripe’ by performing experiments. An integrated sensor suite consisting of ethylene sensor and cameras were installed within the enclosure for quantitative sensing and synchronized recording of the variations in emitted ethylene concentrations and visual degradation characteristics in terms of colour, texture, and firmness of the fruits at a periodic interval of 12 h as shown in Fig. 1a. The ethylene and CO_2_ sensor were procured from Zhengzhou Winsen Electronics Technology Co., Ltd.

Further, a handheld device was developed with ethylene and temperature sensors and a high-resolution camera for measuring ethylene emission and capture images of fruits in real-time under ambient conditions in retail outlets and supermarkets and the same is depicted in Fig. 1b. The handheld device was placed on top of a transparent plastic beaker with the fruit inside to record the variation in ethylene rate and images over a span of 12 h as shown in Fig. 1c. Multiple such installations were used for accelerated experimental analysis.

Fruits used for data annotations for training and testing the predictive models were procured directly from the orchards in Maharashtra (details of orchards mentioned in the “Materials” section). For accurate data labelling, post-harvest respiration and ethylene profile of these fruits were continuously monitored since harvest. The presence or number of micro-organisms were not considered for the scope of our study. Further, to check the test accuracy of these predictive models in real life supply chain scenarios where mixed cultivars of fruits are present, fruits were procured from the Hadapsar Mandai at Pune, Maharashtra. Ripening in climacteric fruits such as banana, mango and papaya is directly proportional to the increase in emission of ethylene concentration and is optimally ripe when the emission of ethylene rate peaks. This phenomenon of ripening in climacteric fruits was used to label the fruits into ‘unripe’ and ‘ripe’. All these fruits considered for experimentation were kept in the enclosure under ambient conditions one at a time post-harvest to see if the ethylene emission rate from the fruits increases over time. Fruit images were labelled as ‘unripe’ when ethylene concentration was found increasing and until the emission of ethylene rate reaches the peak. Fruit images were labelled ‘ripe’ when the ethylene emission was at the peak and ‘overripe’ when the emission rate starts to decrease.

Lemons being non-climacteric fruit are optimally ripe when plucked from plants and do not ripen post-harvest. Like in the case of all non-climacteric fruits, the ethylene emission rate starts to decrease in case of lemons once harvested and the same was observed via experimental analysis. Further, the handheld device was used to observe the trend of increasing ethylene rate over time in the case of pre-harvest lemons which were labelled as ‘unripe’.

### Theoretical methods

Ethylene rate was calculated from the change in naturally emitted ethylene concentrations from fruits over a periodic interval of 12 h as per the below equation:

1 $$ {\text{R }} = [({\text{G}}_{{\text{t}}} - {\text{G}}_{{{\text{t}} - 1}} )/\Delta {\text{t}}]{\text{ V/W}} $$

where, R is the ethylene emission rate, G_t_ and G_t−1_ are the ethylene concentrations at time ‘t’ and ‘t−1’ respectively, V is the volume of the custom-designed enclosure, W is the weight of the fruit^5^. The volume of the custom-designed enclosure was 40 L and volume of the multiple transparent beakers used are 600 mL, 1 L and 2 L and 5 L. Average weight of each papaya was 0.523 kg, each mango was 257 g, each banana of 180 g and each lemon of 35 g.

### Computational methods

#### Transfer learning schematic for climacteric fruits

**Figure 2:**
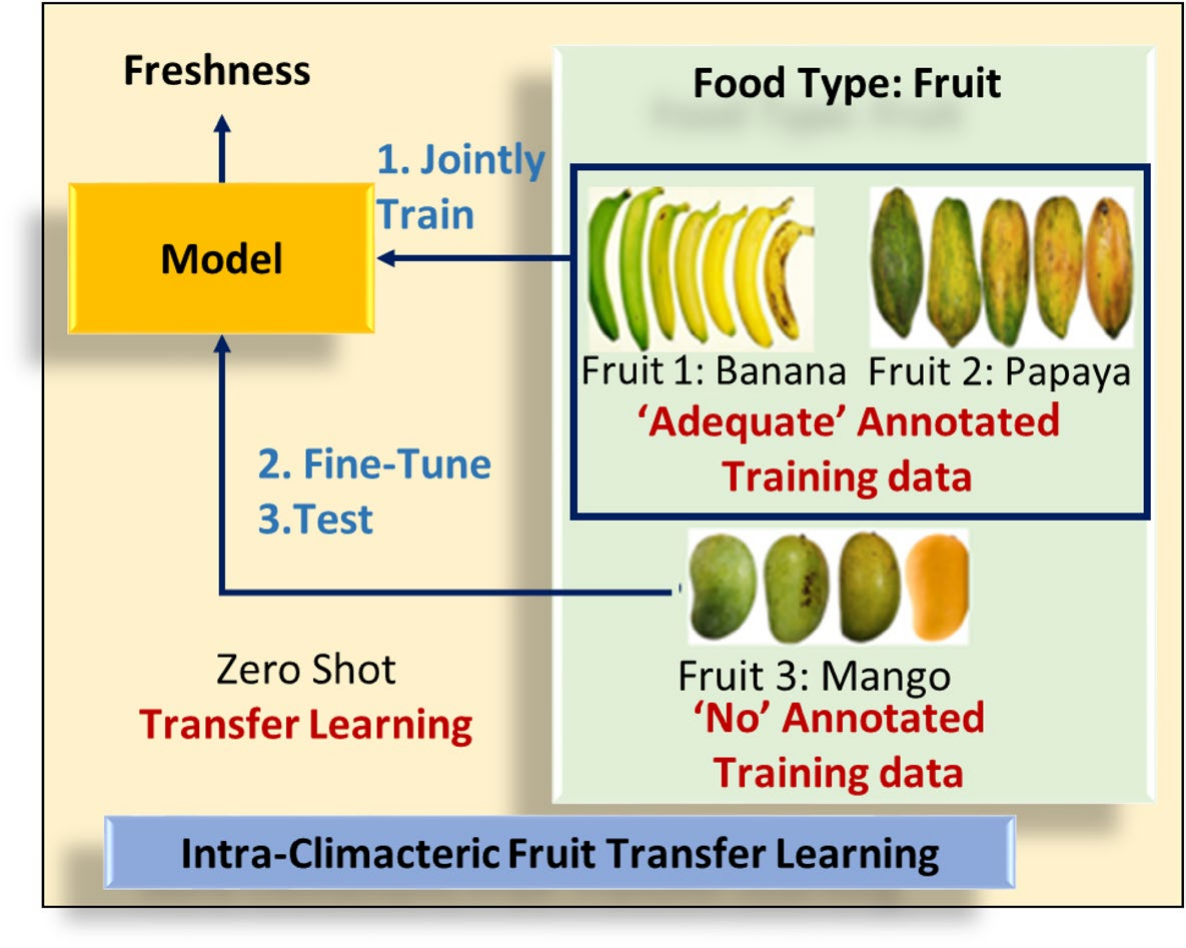
Zero-shot transfer learning schematic for transfer of knowledge within climacteric fruits.

All climacteric fruits ripen post-harvest with an increase in and emission of ethylene concentrations. They are fully ripe when the ethylene emission rate and CO_2_ respiration rate are at the peak and finally start to over ripen with the decrease in both^5^. Zero-shot transfer learning^23^ was used to see if this knowledge of physico-chemical degradation phenomenon is transferable from one climacteric fruit to another. The zero-shot transfer learning architecture used for the transfer of knowledge within climacteric fruits is depicted in Fig. 2. As depicted in Fig. 2, if a CNN model is trained on adequate data of two climacteric fruits for prediction of ripeness level, can the model predict the ripeness level of a third climacteric fruit with good accuracy and without any data of the same as the training set? Also, it was tested that if a CNN model is trained on adequate data of two climacteric fruits for prediction of ripeness level, can it predict the ripeness level of a non-climacteric fruit with good accuracy and without any data of the same in the training set? In our case, adequate experimentally labelled data are available for climacteric fruits namely banana, mango and papaya. Zero-shot transfer learning was implemented on apple, peach, avocado, pear, lemon, litchi, strawberry, cherry and orange. Transfer learning was employed to overcome the lack of (adequate) annotated dataset for all fruits.

#### Data distribution

The image data distributions of fruits used for training the CNN models are tabulated in Table 1. The image datasets for banana, mango, papaya and lemon were labelled as ‘unripe’ and ‘ideally-ripe’ based on experimental analysis. These datasets were divided into training, validation and test datasets in 70:20:10 ratio and were uniformly distributed into two classed ‘unripe and ‘ideally-ripe’. The ‘Fruit-360’ dataset^28^ consisting of images of 120 fruits was taken from ‘Kaggle’ and was divided into training, validation and test datasets in 70:20:10 ratio. The other climacteric fruits namely apple, peach, pear and avocado and non-climacteric fruits namely orange, strawberry, cherry and litchi only have test datasets of 20 samples each and were not used for training the models.

**Table 1:** Data distribution of fruits used for training the CNN models.

Datasets	Classes	Training	Validation	Test
Fruit-360	120	63,338	18,096	9048
Banana	2	290	72	30
Mango	2	290	72	30
Papaya	2	290	72	30
Banana and Papaya	2	580	144	60
Mango and Banana	2	580	144	60
Mango and Papaya	2	580	144	60
Lemon	2	140	40	20
Apple	2	–	–	20
Peach	2	–	–	20
Pear	2	–	–	20
Avocado	2	–	–	20
Orange	2	–	–	20
Strawberry	2	–	–	20
Cherry	2	–	–	20
Litchi	2	–	–	20

#### Model architecture

Weights of deep convolutional neural networks, VGG-16^22^ trained on natural image dataset ‘Imagenet’, without the top fully connected layer was considered for further fine-tuning. The pretrained CNN network VGG-16^22^ performed significantly better with an improved classification accuracy of ripeness levels on test data as compared to other classification methods as they exploit hand-crafted features resulting in limited performance^29^. The pre-trained VGG-16 model was fine-tuned by training all the layers of the model. A ‘FLATTEN’ layer was added to the model output, followed by a ‘DENSE’ fully connected layer of dimension 4096 and activation ‘RELU, a ‘DROPOUT’ of 0.5, followed by another ‘DENSE’ layer of dimension 512 and activation ‘RELU’ and followed by a ‘DROPOUT’ of 0.5. Finally, a ‘DENSE’ fully connected layer was added and trained on 120 classes of fruits from Kaggle ‘Fruit-360’ using activation ‘softmax’ and generate a ‘Fruits’ model, fine-tuned on the aVGG-16 network. The ‘Fruits’ network was trained using ‘Categorical cross-entropy’ loss and ‘SGD’ optimizer^30^ with a momentum of 0.9, learning rate of 0.0001 and a batch size of 8. The ‘Fruits’ model was further fine-tuned by training the last layer on banana, mango, papaya and lemon datasets individually or in combination to develop multiple models to predict ‘unripe’ and ‘ripe’ levels of different climacteric and non-climacteric fruits.

**Figure 3:**
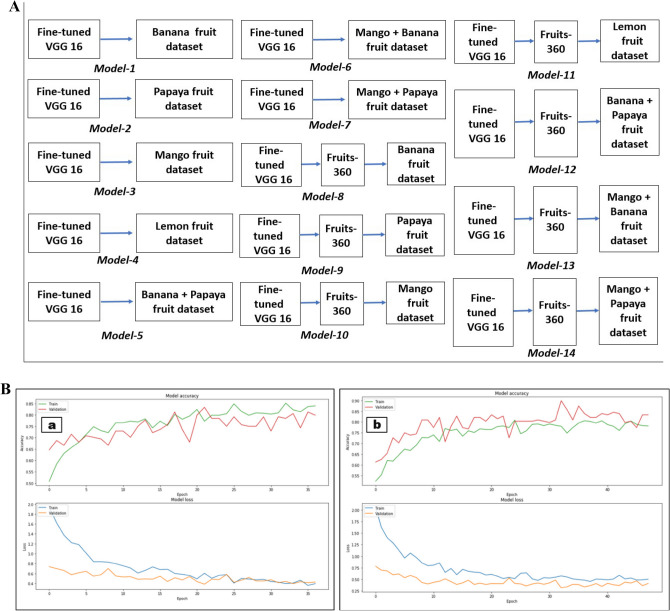
(**A**) The training pipeline for different models used. (**B**) Classification accuracy of ripeness levels on training (**a**) Model 8, (**b**) Model-12.

Fine-tuned VGG-16 network was further fine-tuned on individual fruits dataset namely banana, papaya, mango and lemon to form ‘Model-1’, ‘Model-2’, ‘Model-3’ and ‘Model-4’ respectively. Also, fine-tuned VGG-16 network was further fine-tuned on combined datasets of banana and papaya; mango and banana; and mango and papaya to generate ‘Model-5’; Model-6; and ‘Model-7’respectively. Further, ‘Fruits’ model fine-tuned on VGG-16 network was further fine-tuned on banana; papaya; mango; and lemon datasets to form ‘Model-8’; ‘Model-9’; ‘Model-10’; and ‘Model-11’ respectively. Finally, ‘Fruits’ model fine-tuned on the VGG-16 network was further fine-tuned on combined datasets of banana and papaya; mango and banana; and mango and papaya to generate ‘Model-12’; Model-13; and ‘Model-14’ respectively. The training pipeline for these 13 models is depicted in Fig. 3A. For each of these models, the training and validation datasets were augmented by random rotation, zoom, height shift, width shift, horizontal flip and vertical flip transformations. VGG16 network pretrained on ‘imagenet’ dataset is fine fine-tuned on Fruits-360 dataset followed by individual fruits dataset for better fruit domain adaptation. Data augmentation dramatically expanded training and validation image dataset helped in avoiding data over-fitting and improved robustness of CNN network for classification of ripeness level of fruits. Each of these networks was trained using ‘binary cross-entropy’ loss and ‘SGD’ optimizer^30^ with a momentum of 0.9, a learning rate of 0.0001 and a batch size of 4. All these models were trained for bi-classification of ripeness levels of climacteric and non-climacteric fruits into ‘unripe’ and ‘ripe’ levels and their classification accuracy on test datasets was compared. The developed models were used for zero-shot transfer learning on other fruits namely orange, apple, peach, pear, avocado, lemon, strawberry, litchi and cherry which were not present on the training dataset. The transfer learned accuracies were compared against different models and various inferences were drawn related to the physico-chemical degradation properties of climacteric and non-climacteric fruits.

During the training of each model, the original input images were cropped and resized to the target input size of [224 × 224 × 3] with scale and aspect ratio augmentation^22,23^. The training mechanism involved using the pre-trained VGG-16 model to develop a binary image classifier for classifying climacteric and non-climacteric fruits into ‘unripe’ and ‘ideally-ripe’. The open-source Tensor Flow^31^ was used to implement and train the models on GPU (8 Tesla V100 GPUs, 8 × 32 GB GPU Memory). The models were trained over a training dataset for several epochs with early stopping criteria by monitoring the variation in validation loss. The early stopping criteria specified that if the validation loss no longer decreases below 0.1 after 10 epochs, the training terminates automatically. The variation in training and validation accuracy; and training and validation loss; until the early stopping criteria were met for training two of the above models namely Model-8 and Model-12 are shown in Fig. 3B(a),(b) respectively.

## Results and discussions

### Experimental results

**Figure 4:**
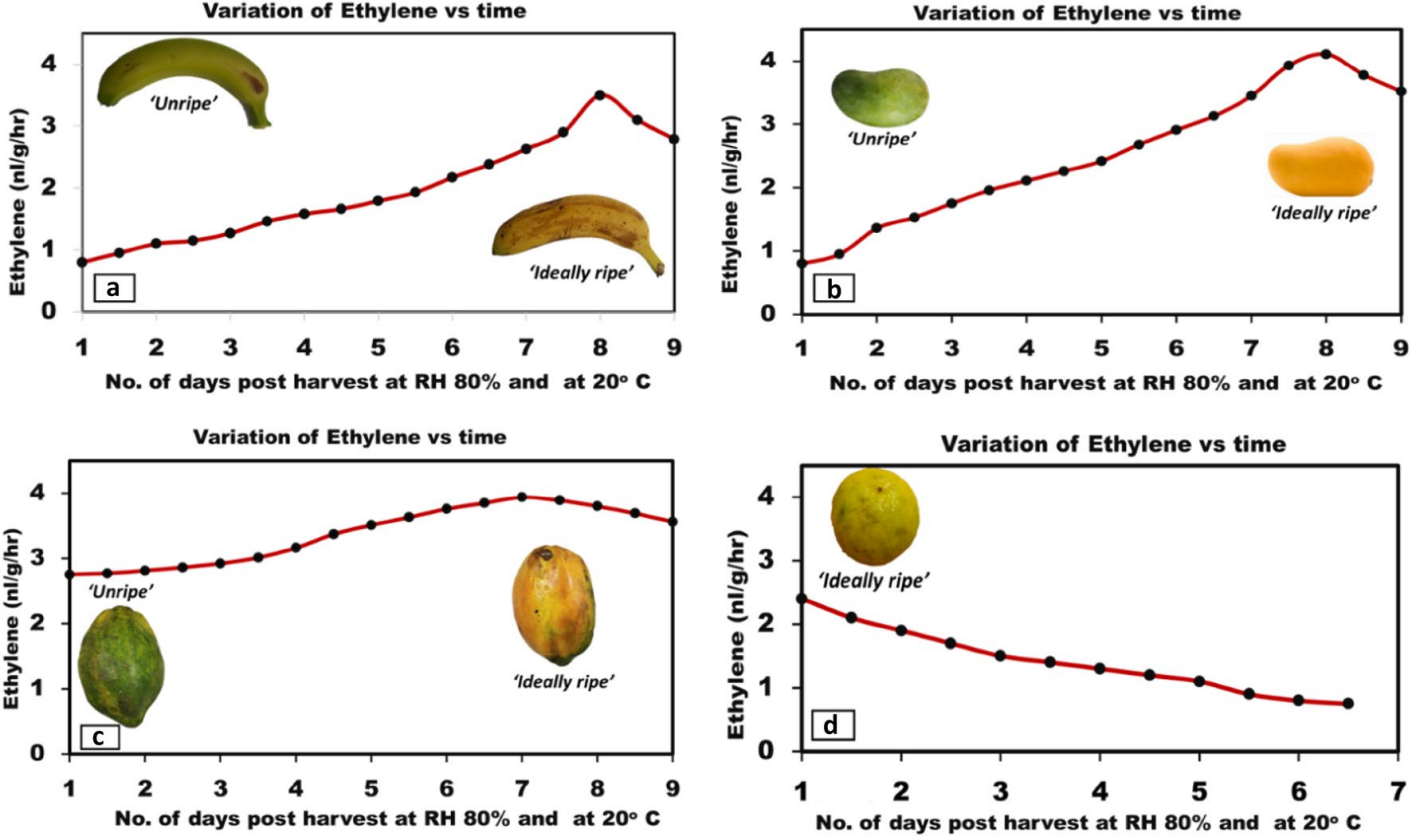
Post-harvest variation of ethylene rate over time at ambient conditions of 20 °C and 80% RH for (**a**) banana, (**b**) mango, (**c**) papaya and (**d**) lemon.

Post-harvest fruit samples namely mangoes, bananas, papayas and lemons used for experimentation were kept in the enclosure and transparent beakers and variation in ethylene rate and images over time were captured at ambient temperature and humidity conditions of 20 °C and 80% RH respectively and one set of such experimental analysis for each fruit is presented in Fig. 4. The climacteric fruits namely banana, mango and papaya were labelled ‘unripe’ till the increase of emission of ethylene rate and were labelled ‘ripe’ when ethylene emission rate was at the peak.

As shown in Fig. 4a, hard green post-harvest bananas with the increase in ethylene rate from 0.8 to 3.5 nL/g/h were labelled as ‘unripe’. As the ethylene emission rate peaked at 3.5 nL/g/h, bananas turned soft and yellow with minor brown spots and best taste and flavour and were then labelled as ‘ripe’.

Similarly, as shown in Fig. 4b, ethylene emission rate was more during the ripening of mangoes as compared to that of bananas and they were labelled as ‘unripe’ when the ethylene emission rate was increasing from 0.8 to 4.11 nL/g/h and were labelled as ‘ripe’ when the ethylene emission rate peaked to 4.11 nL/g/h. Mangoes were hard and green when unripe and turned to soft, juicy and yellow in colour with a strong aroma when ‘ripe’.

Also, as shown in Fig. 4c, hard green papayas were labelled as ‘unripe’ when increasing ethylene emission rate ranged from 2.75 to 3.94 nL/g/h and ‘ripe’ as the ethylene emission rate peaked at 3.94 nL/g/h turning the papayas to yellow in colour and soft in texture.

The variation of ethylene rate over time was also captured for the non-climacteric fruit lemon and the same is presented in Fig. 4d. However, as per the phenomenon of non-climacteric fruits, the ethylene emission rate decreased over time post-harvest. Ethylene emission rate was maximum of the order of 2.4 nL/g/h when plucked from the plant and gradually decreased over time and the same is presented graphically in Fig. 4d. Thus, it was seen that like any other non-climacteric fruits, lemons do not ripen post-harvest and are fully ripe, yellow in colour and soft in texture when plucked and were thereby labelled as ‘ripe’ once harvested.

### Computational results

#### Prediction accuracies of CNN models

The test accuracies (TA) on the classification of fruits into ‘unripe’ and ‘ripe’ levels are presented in Table 2. Fine-tuned VGG-16 model trained on banana (Model-1), papaya (Model-1), mango (Mango-3) and lemon (Mango-4) datasets showed test accuracies of 66.7, 70.7, 72.3 and 79.6 respectively.

**Table 2:** Test accuracy of climacteric fruits using distinct models.

Base model	Trained on	Tested on	Model name	Test accuracy (TA)
Fine-tuned VGG-16	Banana	Banana	Model-1	66.7
Fine-tuned VGG-16	Papaya	Papaya	Model-2	70.7
Fine-tuned VGG-16	Mango	Mango	Model-3	72.3
Fine-tuned VGG-16	Lemon	Lemon	Model-4	79.6
Fine-tuned VGG-16	Banana and papaya	Banana and papaya	Model-5	78.1
Fine-tuned VGG-16	Mango and banana	Mango and banana	Model-6	80.2
Fine-tuned VGG-16	Mango and papaya	Mango and papaya	Model-7	83.3
Fine-tuned VGG-16 further fine-tune on ‘Fruits 360’ dataset	Banana	Banana	Model-8	83.3
Fine-tuned VGG-16 further fine-tune on ‘Fruits 360’ dataset	Papaya	Papaya	Model-9	87.6
Fine-tuned VGG-16 further fine-tune on ‘Fruits 360’ dataset	Mango	Mango	Model-10	84.6
Fine-tuned VGG-16 further fine-tune on ‘Fruits 360’ dataset	Lemon	Lemon	Model-11	86.4
Fine-tuned VGG-16 further fine-tune on ‘Fruits 360’ dataset	Banana and papaya	Banana and papaya	Model-12	87.5
Fine-tuned VGG-16 further fine-tune on ‘Fruits 360’ dataset	Mango and banana	Mango and banana	Model-13	88.2
Fine-tuned VGG-16 further fine-tune on ‘Fruits 360’ dataset	Mango and papaya	Mango and papaya	Model-14	89.9

When the datasets of two fruits were combined and fine-tuned VGG-16 network was trained on the combined fruits dataset, the test accuracies increased as compared to fine-tuned VGG-16 network trained on the individual fruit dataset. When fine-tuned VGG-16 network was trained on a combined dataset of banana and papaya (Model-5); mango and banana (Model-6); and mango and papaya (Model-7), the test accuracies were 78.1, 80.1 and 83.3 respectively. Thus, in case of Model-5, TA is 78.1 which greater than 66.7 and 70.7 implying that TA is improved when fine-tuned VGG-16 is trained on a combined dataset of banana and papaya in comparison to individual datasets of banana and papaya. Similarly, in case of Model-6, TA is 80.1 which is greater than 72.3 and 66.7 implying that TA is improved when fine-tuned VGG-16 is trained on a combined dataset of mango and banana in comparison to individual datasets of mango and banana. Also, in case of Model-7, TA is 83.3 which is greater than 72.3 and 70.7 implying that TA is improved when fine-tuned VGG-16 is trained on a combined dataset of mango and papaya in comparison to individual datasets of mango and papaya.

Further, when fine-tuned VGG-16 network was first fine-tuned on the ‘Fruit-360’ dataset (‘Fruits’) and then trained on the individual or combined fruits dataset, the test accuracies improved significantly as compared to the above models. ‘Fruit-360’ dataset consists of 120 classes of fruits including banana, papaya and mango. Fine-tuned VGG-16 network first fine-tuned on ‘Fruits’ network and further fine-tuned on banana (Model-8), papaya (Model-9), mango (Model-10) and lemon (Model-11) datasets showed TAs of 83.3, 87.6, 84.6 and 86.4 respectively. As shown in Table 2, Model-8 showed TA of 83.3 which is greater than TA of 66.7 showed by Model-1. Similarly, Model-9, Model 10 and Model-11 showed PA of 87.6, 84.6 and 86.4; which is greater than TA of 70.7, 72.3 and 79.6 showed by Model-2, Model-3 and Model 4 respectively.

When fine-tuned VGG-16 network was first fine-tuned on ‘Fruits’ network and further fine-tuned on the combined fruits dataset, there were significant improvements on the TAs in comparison to all the other models. Fine-tuned VGG-16 network first fine-tuned on ‘Fruits’ network and further fine-tuned on combined datasets of banana and papaya (Model-12); mango and banana (Model-13); and mango and papaya (Model-14), the test accuracies were 87.5, 88.2 and 89.9 respectively. As shown in Table 2, Model-12 showed TA of 87.5 which is greater than TA of 66.7, 70.7 and 78.1 showed by Model-1, Model-2 and Model-5 respectively. Similarly, Model-13, showed TA of 88.2 which is greater than TA of 72.3, 66.7 and 80.2 showed by Model-3, Model-1 and Model-6 respectively. Also, Model-14 showed TA of 89.9 which is greater than 72.3, 70.7 and 83.3 showed by Model-3, Model-2 and Model-7 respectively. Thereby, it can be concluded that fine-tuning of the VGG-16 network on the ‘Fruit-360’ dataset was significantly beneficial in improving the test accuracies on test dataset of fruits.

#### Transfer learned accuracies

Fine-tuned VGG-16 network trained on banana dataset (Model-1) to classify ripeness levels into ‘unripe’ and ‘ripe’, was used for zero-shot transfer learning (TL) on papaya, mango and lemon without any of their data being present on the training set. Similarly, fine-tuned VGG-16 network trained on papaya (Model-2); mango (Model-3); and lemon (Model-4); datasets were used for TL on banana, mango and lemon; banana, papaya and lemon; and banana, papaya and mango respectively and the same is shown in Table 3. Model-2 and Model-3 without any image of banana showed zero-shot transfer learned accuracies of 56.9 and 63.4 on banana respectively which are comparable to the test accuracy (TA) of 66.7 obtained by training VGG-16 network on banana data (Model-1). Model-1 and Model-3 without any image of papaya showed zero-shot transfer learned accuracies of 62.6 and 65.3 on papaya respectively which are comparable to the TA of 70.7 obtained by training VGG-16 network on papaya data (Model-2). Model-1 and Model-2 without any image of mango showed zero-shot transfer learned accuracies of 66.7 and 64.8 on mango respectively which are comparable to the TA of 72.3 obtained by training VGG-16 network on mango data (Model-3). The zero-sot transfer learning was successful in case of banana, mango and papaya and this might be because of their similarity in ripening characteristics and changes of physical attributes like colour change from green to yellow and firmness change from hard to soft. This implies a successful transfer of knowledge from one climacteric fruit to another which is per the similarity of physico-chemical degradation phenomenon of climacteric fruits.

**Table 3:** Transfer Learned accuracies for different CNN networks. *The test accuracy (TA) indicates accuracies on test dataset of fruits by using the corresponding model as shown in Table 3 which is trained on the same fruits. For more details on TA Table 2 can be referred.

Model Name	Test accuracy (TA)*	TL on banana	TL on papaya	TL on mango	TL on lemon
Model-1	66.7	–	62.6	66.7	10
Model-2	70.7	56.9	–	64.8	9.6
Model-3	72.3	63.4	65.3	–	15
Model-4	79.6	7.6	20.6	14.7	–
Model-5	78.1	68.6	71.2	73.3	15
Model-6	80.2	68.9	71.4	73.2	12.8
Model-7	83.3	70.3	71.7	72.6	22
Model-8	83.3	–	72.6	73.9	18
Model-9	87.6	73.7	–	76.4	16.7
Model-10	84.6	76.7	78.7	–	28.6
Model-11	86.4	16.8	31.7	26.5	–
Model-12	87.5	85.6	86.1	84.3	23.6
Model-13	88.2	85.2	81.2	85.5	20.8
Model-14	89.9	79	86.7	86.3	32.3

However, Model-1, Model-2 and Model-3 without any image of lemon showed zero-shot transfer learned accuracies of 10, 9.6 and 15 on lemon respectively which are very less as compared to the TA of 79.6 obtained by training VGG-16 network on lemon data (Model-4). This implies that transfer learning was not successful for the transfer of knowledge from climacteric fruits namely banana, mango and papaya to non-climacteric fruit lemon. Also, Model-4 without any image of banana, papaya and mango showed zero-shot transfer learned accuracies of 7.6, 20.6 and 14.7 on banana, papaya and mango respectively which are not comparable to the TA of 66.7, 70.7 and 72.3 obtained by training VGG-16 network on banana (Model-1), papaya (Model-2) and mango datasets (Model-3) respectively. Further, all the other models listed in Table 3 trained on ‘Fruits’ network and further fine-tuned on individual or combined dataset of climacteric fruits namely banana, mango and papaya when used for zero-shot TL on non-climacteric fruit lemon, or vice-versa, the TAs were not exemplary. The reason for this is lemon is a non-climacteric fruit which doesn’t show significant visual degradation characteristics unlike the climacteric fruits which shows similar visual changes in terms of color, black spots, molds, fungus and rate of degradation. Our models worked better for climacteric fruits as compared to non-climacteric fruits and hence TL accuracy on lemon was lower than other climacteric fruits. This further proves that due to the dissimilarity in post-harvest degradation mechanism of climacteric and non-climacteric fruits transfer of knowledge was not successful from climacteric to non-climacteric fruits and vice-versa.

Fine-tuned VGG-16 network trained on a combined dataset of banana and papaya (Model-5) showed zero-shot transfer learned accuracy of 73.3 on mango without any data of mango being present on the training set. Similarly, Model-6 and Model-7 showed zero-shot transfer learned accuracy of 71.4 on papaya and 70.3 on banana respectively. TA of 73.3 on mango obtained from Model-5 is greater than zero-shot transfer learned accuracies of 66.7 and 64.8 on mango obtained from Model-1 and Model-2 respectively implying improvement in transfer learned test accuracy on combining the training dataset of banana and papaya. Zero-shot TL accuracy of 71.4 on papaya obtained from Model-6 is greater than zero-shot transfer learned accuracy of 62.6 and 65.3 on papaya obtained from Model-1 and Model-3 respectively. Zero-shot TL accuracy of 70.3 on banana obtained from Model-7 is greater than zero-shot transfer learned accuracy of 56.9 and 63.4 on banana obtained from Model-2 and Model-3 respectively. From these observations, it is thus implied that there is improvement in transfer learned accuracy on combining the training dataset of two climacteric fruits.

Also, Model-5 showed TL accuracies of 68.6 and 71.2 on banana and papaya test set which was greater than the TA of 66.7 and 70.7 obtained by training VGG-16 network on banana data (Model-1) and papaya data (Model-2) respectively. Similarly, Model-6 showed TL accuracies of 68.9 and 73.2 on banana and mango test set which was greater than the TA of 66.7 and 72.3 obtained by training VGG-16 network on banana data (Model-1) and mango data (Model-3) respectively. Further, Model-7 showed TL accuracies of 72.6 and 71.7 on mango and papaya test set which was greater than the TA of 72.3 and 70.7 obtained by training VGG-16 network on mango data (Model-3) and papaya data (Model-2) respectively. This further implies that combining the datasets of two climacteric fruits namely banana and papaya or mango and banana or mango and papaya helped in improving the test accuracies of individual fruits.

Fine-tuned VGG-16 network when first fine-tuned on ‘Fruits’ network and further fine-tuned on banana (Model-8) showed zero-shot transfer learned accuracies of 72.6 and 73.9 on papaya and mango respectively. Zero-shot TL accuracies of 72.6 and 73.9 obtained from Model-8 are greater than zero-shot transfer learned accuracies of 62.6 and 66.7 on papaya and mango respectively obtained from Model-1. Similarly, Model-9 showed zero-shot transfer learned accuracies of 73.7 and 76.4 on banana and mango respectively. Zero-shot TL accuracies of 73.7 and 76.4 obtained from Model-9 are greater than zero-shot transfer learned accuracies of 56.9 and 64.8 on banana and mango respectively obtained from Model-2. Further, Model-10 showed zero-shot transfer learned accuracies of 76.7 and 78.7 on banana and papaya respectively. Zero-shot TL accuracies of 76.7 and 78.7 obtained from Model-10 are greater than zero-shot transfer learned accuracies of 63.4 and 65.3 on banana and papaya respectively obtained from Model-3. All these results implied that an intermediate ‘Fruits’ network helped in the improvement of zero-shot transfer learned accuracies of climacteric fruits.

Fine-tuned VGG-16 network when first fine-tuned on ‘Fruits’ network and further fine-tuned on a combined dataset of banana and papaya (Model-12); mango and banana (Model-13); mango and papaya (Model-14) showed zero-shot transfer learned accuracies of 84.3 on mango; 81.2 on papaya and 79 on banana respectively. Zero-shot TL accuracy of 84.3 on mango obtained from Model-12 is greater than zero-shot transfer learned accuracies of 66.7, 64.8, 73.3, 73.9 and 76.4 on mango obtained from Model-1, Model-2, Model-5, Model-8 and Model-9 respectively. This implied that there is a significant improvement in transfer learned accuracy on first fine-tuning the VGG-16 model on a ‘Fruits’ network followed by fine-tuning on a combined dataset of banana and papaya. Similarly, zero-shot TL accuracy of 81.2 on papaya obtained from Model-13 is greater than zero-shot transfer learned accuracies of 62.6, 65.3, 71.4, 72.6 and 78.7 on papaya obtained from Model-1, Model-3, Model-6, Model-8 and Model-10 respectively. Further, zero-shot TL accuracy of 79 on banana obtained from Model-14 is greater than zero-shot transfer learned accuracies of 56.9, 63.4, 70.3, 73.7 and 76.7 on banana obtained from Model-2, Model-3, Model-7, Model-9 and Model-10 respectively. These results implied that there is a significant improvement in transfer learned prediction accuracy than all the above-mentioned scenarios on first fine tuning the VGG-16 model on a ‘Fruits’ network followed by fine-tuning on a combined dataset of two climacteric fruits.

Model-12 and Model-13 resulted in zero-shot TL accuracy of 85.6 and 85.2 respectively on banana test dataset which was greater than TA of 66.7 and 83.3 obtained by using Model-1 and Model-8 respectively and zero-shot TL accuracies of 56.9, 63.4, 68.6, 68.9, 70.3, 73.7 and 76.7 by using Model-2, Model-3, Model-5, Model-6, Model-7, Model-9 and Model-10 respectively on test dataset of banana, implying a significant improvement in the prediction of ripeness level of banana by fine-tuning VGG-16 network on ‘Fruits’ dataset followed by a combined dataset of two climacteric fruits namely banana and papaya; and mango and banana.

Similarly, Model-12 and Model-14 resulted in zero-shot TL accuracies of 86.1 and 86.7 respectively on papaya test dataset which were greater than TA of 70.7 and 87.6 obtained by using Model-2 and Model-9 respectively and zero-shot TL accuracies of 62.6, 65.3, 71.2, 71.4, 71.7, 72.6 and 78.7 by using Model-1, Model-3, Model-5, Model-6, Model-7, Model-8 and Model-10 respectively on test dataset of papaya implying a significant improvement in the prediction of ripeness level of papaya by fine-tuning VGG-16 network on ‘Fruits’ dataset followed by a combined dataset of two climacteric fruits namely banana and papaya; and mango and papaya.

Further, Model-13 and Model-14 resulted in zero-shot TL accuracies of 85.5 and 86.3 respectively on mango test dataset which were greater than TAs of 72.3 and 84.6 obtained by using Model-3 and Model-10 respectively and zero-shot TL accuracies of 66.7, 64.8, 73.3, 73.2, 72.6, 73.9 and 76.4 by using Model-1, Model-2, Model-5, Model-6, Model-7, Model-8 and Model-9 respectively on test dataset of mango, implying a significant improvement in the prediction of ripeness level of mango by fine-tuning VGG-16 network on ‘Fruits’ dataset followed by a combined dataset of two climacteric fruits namely mango and banana; and mango and papaya.

Thus, Model-12, Model-13 and Model-14 resulted in the best test accuracies for the prediction of ripeness level of climacteric fruits banana, papaya and mango. These models were further used for the prediction of ripeness levels of unknown samples of climacteric fruits namely apple, peach, pear and avocado and non-climacteric fruits namely Orange, Cherry, Litchi and Strawberry by zero-shot transfer learning technique. The Transfer Learned accuracies on these unknown samples of climacteric and non-climacteric fruits were not present in the training dataset of Model-12, Model-13 and Moel-14 and the same is presented in Table 4.

**Table 4:** Transfer learned accuracies on unknown samples of climacteric and non-climacteric fruits for different CNN networks.

Model name	Test accuracy (TA)	TL on apple	TL on peach	TL on pear	TL on avocado	TL on orange	TL on cherry	TL on litchi	TL on strawberry
Model-12	87.5	71.9	72.6	79.6	65.8	35.4	26.8	35.7	24.8
Model-13	88.2	74.6	70.9	82.4	56.9	45.9	35.6	28.6	27.6
Model-14	89.9	80.9	76.9	80.3	68.1	38.7	47.6	39.3	36.5

Model-12 resulted in zero-shot transfer learned accuracies of 71.9, 72.6, 79.6, 65.8, 35.4, 26.8, 35.7 and 24.8 on apple, peach, pear, avocado, orange, cherry, litchi and strawberry respectively. Model-13 resulted in zero-shot transfer learned accuracies of 74.6, 70.9, 82.4, 56.9, 45.9, 35.6, 28.6 and 27.6 on apple, peach, pear, avocado, orange, cherry, litchi and strawberry respectively. Model-14 resulted in zero-shot transfer learned accuracies of 80.9, 76.9, 80.3, 68.1, 38.7, 47.6, 39.3 and 36.5 on apple, peach, pear, avocado, orange, cherry, litchi and strawberry respectively. Thus, Model-12, Model-13 and Model-14 trained on climacteric fruits namely banana, papaya and mango resulted in satisfactory zero-shot transfer learned accuracies in the range of 70 to 82 for climacteric fruits.

Further, it was observed that the zero-shot transfer learned accuracy was less for avocado as compared to other climacteric fruits namely, apple, peach and pear. The reason for the same can be attributed to the fact that avocado has a complex ripening process with very less sugar content of about 0.7 g in 100 g by weight. Model-12, Model-13 and Model-14 are trained on climacteric fruits banana, mango and papaya with high sugar content in the range of 8–14 g in 100 g by weight^32–34^. These models when used for zero-shot transfer learning on climacteric fruits, the prediction of ripeness level was satisfactory for fruits with higher sugar content namely apple, peach and pear in the range of 10 to 13 g in 100 g by weight unlike avocado^32–34^. Thus, we can further conclude that these models result in better predictions for fruits with higher sugar content in comparison to fruits with lower sugar content.

However, these models trained on climacteric fruits when were used for zero-shot transfer learning on non-climacteric fruits, the test accuracies were unsatisfactory and in the range 25 to 48. The reason behind the unsatisfactory result can be attributed to the dissimilarity in the ripening pattern and visual attributes between climacteric and non-climacteric fruits^5^. This further implies that the transfer of knowledge was successful from climacteric to non-climacteric fruits but not from climacteric to non-climacteric fruits.

## Conclusion

This paper presents generic AI models trained on individual or combined dataset of climacteric fruits namely banana, papaya and mango and non-climacteric fruit lemon for prediction of ripeness levels of a variety of climacteric fruits (apple, peach, pear and avocado) and non-climacteric fruits (orange, cherry, litchi and strawberry) by using ‘zero-shot’ transfer learning techniques. The zero-shot transfer learning works better within climacteric fruits due to the similarity in physico-chemical degradation phenomena depicted by their visual properties such as black spot formations, wrinkles, discoloration, etc. Among all the models, VGG-16 network fine-tuned on ‘Fruits’ dataset and further fine-tuned on a combined dataset of climacteric fruits namely banana and papaya (Model-12); mango and banana (Model-13); and mango and papaya (Model-14) showed the best results in prediction of ripeness levels of climacteric fruits, especially with high sugar content. The zero-shot transfer learned accuracies obtained on using Model-12, Model-13 and Model-14 for prediction of ripeness levels of a variety of climacteric fruits and non-climacteric fruits clearly showed that transfer of knowledge was better demonstrated within climacteric fruits as compared to non-climacteric fruits and vice-versa. The results were in sync with the known degradation chemistry of climacteric and non-climacteric fruits. These generic models once deployed, acts as soft-sensor for the prediction of ideal ripeness levels of a variety of climacteric fruits non-invasively in real-time, thus providing a cost-effective solution to the stakeholders of the fruit supply chain in taking dynamic decisions related to repurposing, repricing and rerouting to reduce wastage and maximize profit.

## Data Availability

The datasets used and/or analyzed during the current study are available from the corresponding author on reasonable request.
